# Myoid hamartoma of the breast with focal chondromyoxid metaplasia and pseudoangiomatous stromal hyperplasia: A case report

**DOI:** 10.3892/ol.2015.2892

**Published:** 2015-01-23

**Authors:** CHIN-CHENG SU, CHIH-JUNG CHEN, SHOU-JEN KUO, DAR-REN CHEN

**Affiliations:** 1 Tumor Research Center of Integrative Medicine, Changhua Christian Hospital, Changhua 50006, Taiwan, R.O.C.; 2 Comprehensive Breast Cancer Center, Changhua Christian Hospital, Changhua 50006, Taiwan, R.O.C.; 3 Department of Surgery, Changhua Christian Hospital, Changhua 50006, Taiwan, R.O.C.; 4 School of Chinese Medicine, College of Chinese Medicine, China Medical University, Taichung 40402, Taiwan, R.O.C.; 5 Department of Surgical Pathology, Changhua Christian Hospital, Changhua 50006, Taiwan, R.O.C.; 6 School of Medicine, Chung Shan Medical University, Taichung 40201, Taiwan, R.O.C.; 7 Department of Medical Technology, Jen-Teh Junior College of Medicine, Nursing and Management, Miaoli 356, Taiwan, R.O.C.

**Keywords:** breast myoid hamartoma, chondromyoxid, pseudoangiomatous

## Abstract

Hamartomas of the breast, also known as fibroadenolipomas, lipofibroadenomas or adenolipomas, are benign lesions. Hamartomas account for between 0.04 and 1.15% of all benign breast tumors in females. Myoid hamartoma of the breast (MHB) is extremely rare. The present study describes a case of MHB in a 44-year-old female. Screening mammography revealed a lobulated partial indistinct isodense mass measuring ~3.8 cm in the upper outer quadrant of the left breast. Sonographic examinations revealed a 2–3-cm mass in the left breast, which was fairly well circumbscribed and demonstrated complex scattered echogenic areas and isoechoic tissue. A core needle biopsy demonstrated fibrocystic changes, with small focal ductule aggregations. As malignancy could not be excluded, a partial mastectomy was performed using a circumareolar incision. The mass was histopathologically diagnosed as MHB with focal chondromyoxid metaplasia and pseudoangiomatous stromal hyperplasia. The histological diagnosis was based upon the findings of the well-circumscribed tumor, which was composed of entrapped mammary ducts, fat cells and myoid stromal components, with focal chondromyxoid metaplasia and pseudoangiomatous stromal hyperplasia. The tumor cells exhibited diffuse cluster of differentiation 34-positive immunoreactivity, which was consistent with a diagnosis of pseudoangiomatous stromal hyperplasia.

## Introduction

Hamartomas of the breast, also known as fibroadenolipomas, lipofibroadenomas or adenolipomas, are benign lesions that were first described in 1971 (1). Hamartomas account for between 0.04 and 1.15% of all benign breast tumors in females (2,3). Myoid hamartoma of the breast is composed of differentiated mammary glandular and stromal structures and is considered to be a rare variant of mammary hamartoma. Myoid hamartomas may present as painless breast lumps, using sonography it has been revealed that the majority of hamartomas are hyperechoic or composed of mixed echogenicity, retrotumor acoustic phenomena are absent. Breast hamartomas are rare lesions, that may be misdiagnosed pre-operatively, with a definitive diagnosis made by histological examination. As a hamartoma has the potential to progress to breast cancer (3–10), surgical removal is the curative treatment for breast hamartomas (11,12). The patient provided written informed consent.

## Case report

**Figure 1:**
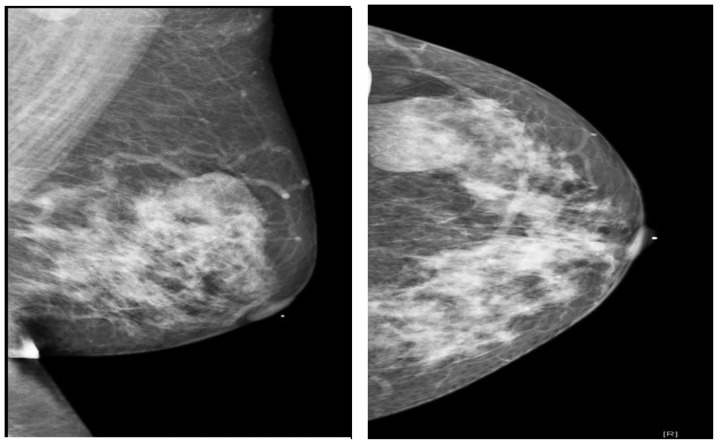
Mammography performed to rule out malignancy revealing an ovoid, well-circumscribed and lobulated partial indistinct isodense mass measuring ~3.8 cm in the upper outer quadrant of the left breast.

**Figure 2:**
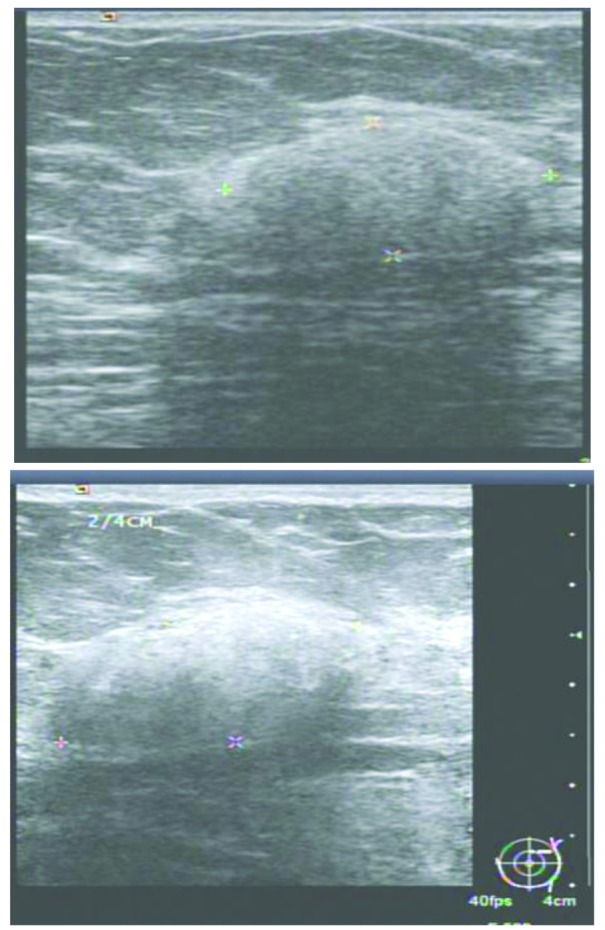
Sonography revealing a 2–3-cm, well-encapsulated mass in the left breast with complex scattered echogenic areas measuring ~2.42×2.17×1.36 cm, which displaced the adjacent normal breast tissue and was consistent with a diagnosis of lipoma and internal inhomogeneity.

**Figure 3:**
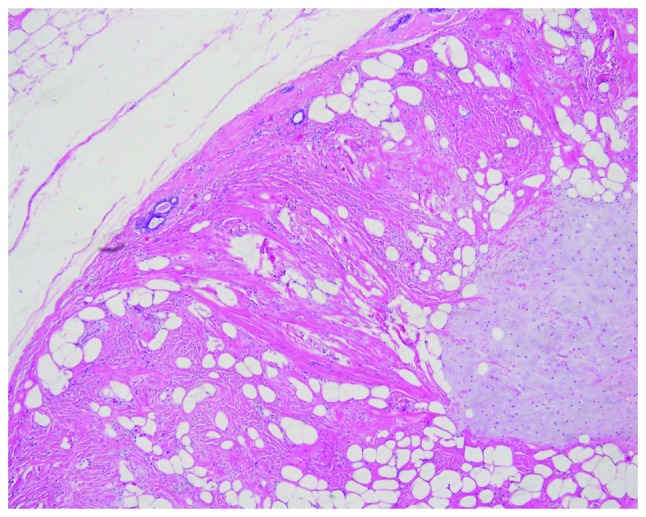
Well-circumscribed tumor composed of entrapped mammary ducts, fat cells and myoid stromal components, with focal chondromyxoid metaplasia and pseudoangiomatous stromal hyperplasia. Hematoxylin and eosin staining; magnification, ×100.

**Figure 4:**
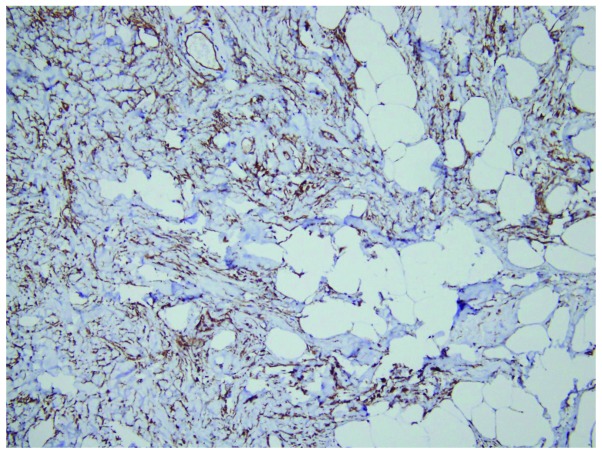
Tumor cells exhibiting diffuse cluster of differentiation 34-positive immunoreactivity, consistent with a diagnosis of pseudoangiomatous stromal hyperplasia. Immunohistochemical staining; magnification, ×200.

A 44-year-old female was admitted to the Changhua Christian Hospital, LuKang branch Hospital, Outpatient Department (Changhua, Taiwan) on March 12, 2014 with a mass in the left breast that had been apparent for four months. A physical examination revealed the presence of a mass measuring ~3 cm in diameter in the upper outer quadrant (UOQ) of the left breast. The presence of a contralateral mass or axillary lymphadenopathy was not evident. Mammographical examinations performed to rule out malignancy revealed a lobulated partial indistinct isodense mass measuring ~3.8 cm in the UOQ of the left breast (Fig. 1) and suggested that further evaluation and tissue examinations were necessary. Ultrasonography examinations revealed a ~2.42×2.17×1.36 cm mass in the left breast, which was fairly well-circumscribed and demonstrated complex scattered echogenic areas and isoechoic tissue, which was consistent with a diagnosis of lipoma (Fig. 2). Due to the presence of heterogeneous echo dense areas, an ultrasound-guided core needle biopsy was recommended for further assessment. The core needle biopsy revealed fibrocystic changes with small focal duct aggregations. Immunohistochemically, intact myoepithelial cells were identified using p63 staining. A diagnosis of focal adenosis was considered. As malignancy could not be excluded, an excisional biopsy was performed using a circumareolar incision. The excised specimen, which consisted of a tissue fragment measuring 5.5×3.4×2.5 cm, was fixed in formalin. Grossly, the tissue appeared tan-white in color and elastic. Upon dissection, a whitish nodule measuring 1.7×1.5 cm was identified. Microscopically, a well-circumscribed tumor measuring 1.9×1.8 cm was identified, which was primarily composed of fibrous stroma, spindle cells, abundant fat cells and scatted mammary ductolobular units in the center or periphery of the tumor. Pseudoangiomatous stromal hyperplasia was also evident in the tumor region. Immunostaining revealed that the spindle cells were p63(−), cluster of differentiation 34(+) and desmin(+). Focal microcalcification was also apparent inside the ducts and lobules. Consequently, a diagnosis of myoid hamartoma with focal chondromyxoid differentiation and pseudoangiomatous stromal hyperplasia was established (Figs. 3 and 4). The patient exhibited no post-operative complications during the Outpatient Department follow-up and no further treatment was necessary.

## Discussion

Myoid hamartomas may present as painless breast lumps that can be misdiagnosed pre-operatively. It is well documented that the internal echo texture of the majority of hamartomas is hyperechoic or composed of mixed echogenicity. Retrotumor acoustic phenomena are absent in the majority of hamartomas (13), however, the phenomena were present in the patient in the present study(Fig. 2). Breast hamartomas are well-circumscribed, solid, oval tumors without intratumor microcalcification. In cases where a benign breast lesion is encountered with microcalcifications, coexistent malignancy should be ruled out (14). The patient in the present study presented with a breast tumor with focal microcalcification inside the ducts and lobules, but with the absence of any malignancy. Breast hamartomas are rare lesions, with a definitive diagnosis made by histological examination. The use of excisional biopsies and immunohistochemical analyses are important in order to avoid confusion during the diagnosis of benign and malignant spindle cell tumors (15,16). It is well documented that the use of fine-needle aspiration cytology and needle biopsy is unlikely to provide sufficient evidence for pathologists (17). In the present study, a core needle biopsy of the breast tumor revealed fibrocystic changes with small focal duct aggregations, in agreement with the literature (17). Although hamartomas are benign, coincidental malignancy may occur. Therefore, surgical removal is the curative treatment for breast hamartomas (11,12). The patient in the present study underwent a partial mastectomy in order to achieve a tumor-free margin. The pathological report established a diagnosis of myoid hamartoma with focal chondromyxoid differentiation and pseudoangiomatous stromal hyperplasia, which is an exceptionally rare breast lesion. Post-operative complications were not observed during the Outpatient Department follow-up.
